# Investigating the Possible Contributions of nsSNPs in ADAM10 and ADAM17 to Alzheimer's Disease Progression: An in Silico Analysis

**DOI:** 10.1002/alz70855_102541

**Published:** 2025-12-23

**Authors:** Muhammet Furkan Açık, Merve Alaylıoğlu

**Affiliations:** ^1^ Institute of Neurological Sciences, Istanbul University‐Cerrahpaşa, Istanbul, Turkey

## Abstract

**Background:**

Alpha‐secretase, a member of the ADAM protein family, cleaves amyloid precursor protein (APP) within the amyloid‐beta (Aβ) domain, preventing Aβ formation, a hallmark of Alzheimer's disease (AD). Alpha‐secretase activity, primarily mediated by *ADAM10* and *ADAM17*, has neuroprotective effects. *ADAM10* and *ADAM17* influence several proteins implicated in AD pathogenesis. For instance, *ADAM10* facilitates the shedding of *TREM2*, which is critical for microglial activation, Aβ clearance, and inflammation control. *ADAM17* modulates the release of tumor necrosis factor‐alpha (TNF‐α), a pro‐inflammatory cytokine linked to neuroinflammation and neuronal damage in AD. Dysregulation in these pathways due to genetic variations or altered enzymatic activity of *ADAM10* and *ADAM17* may exacerbate AD progression. Investigating genetic variations and their effects on enzyme activity is crucial for understanding the broader impact of *ADAM10* and *ADAM17* in AD pathogenesis and identifying potential therapeutic targets. Therefore, in this study, we aimed to identify non‐synonymous single nucleotide polymorphisms (nsSNPs) in *ADAM10* and *ADAM17* and evaluate their potential pathogenicity in silico.

**Method:**

SNPs and protein sequence data for *ADAM10* and *ADAM17* were retrieved from the dbSNP and Ensembl databases. Protein models were obtained from AlphaFold and modeled using Phyre2. The pathogenic potential of identified SNPs was assessed through SIFT, PolyPhen‐2, PhD‐SNP, PredictSNP1/2, Meta SNP, FATHMM, SNPs&GO, and CADD. Structural stability and disease relevance were evaluated with DynaMut2, MutPred, MUpro, mCSM, mCSM‐Membrane, INPS‐MD, and Missense3D, while protein region conservation was analyzed using PANTHER and Consurf.

**Result:**

Initial findings suggest that *rs1596019164, rs200737587* and *rs1896150983* in *ADAM10*, as well as *rs1339437801*, and *rs951262662* in *ADAM17*, may disrupt alpha‐secretase activity, potentially altering Aβ production and contributing to AD progression. These variants could impact protein stability and enzymatic efficiency, further influencing disease mechanisms. Notably, some variants are located in conserved and functionally significant regions, suggesting a stronger pathogenic potential.

**Conclusion:**

The analysis highlights the presence of variants with high pathogenicity scores, suggesting their significant role in disease mechanisms. This study identifies potentially pathogenic nsSNPs in *ADAM10* and *ADAM17* that may affect their alpha‐secretase activity and contribute to AD pathogenesis. Further experimental validation is essential to confirm these results and clarify their roles in disease mechanisms.

